# Effect of Plyometric Training on Handspring Vault Performance and Functional Power in Youth Female Gymnasts

**DOI:** 10.1371/journal.pone.0148790

**Published:** 2016-02-09

**Authors:** Emma Hall, Daniel C. Bishop, Thomas I. Gee

**Affiliations:** School of Sport and Exercise Science, University of Lincoln, Lincoln, United Kingdom; Texas A&M University, UNITED STATES

## Abstract

This study aimed to determine the effect of plyometric training (PT) when added to habitual gymnastic training (HT) on handspring vault (HV) performance variables. Twenty youth female competitive gymnasts (Age: 12.5 ± 1.67 y) volunteered to participate and were randomly assigned to two independent groups. The experimental plyometric training group (PTG) undertook a six-week plyometric program, involving two additional 45 min PT sessions a week, alongside their HT, while the control group (CG) performed regular HT only. Videography was used (120 Hz) in the sagittal plane to record both groups performing three HVs for both the baseline and post-intervention trials. Furthermore, participants completed a countermovement jump test (CMJ) to assess the effect of PT on functional power. Through the use of Quintic biomechanics software, significant improvements (*P* < 0.05) were found for the PTG for run-up velocity, take-off velocity, hurdle to board distance, board contact time, table contact time and post-flight time and CMJ height. However, there were no significant improvements on pre-flight time, shoulder angle or hip angle on the vault for the PTG. The CG demonstrated no improvement for all HV measures. A sport-specific PT intervention improved handspring vault performance measures and functional power when added to the habitual training of youth female gymnasts. The additional two hours plyometric training seemingly improved the power generating capacity of movement-specific musculature, which consequently improved aspects of vaulting performance. Future research is required to examine the whether the improvements are as a consequence of the additional volume of sprinting and jumping activities, as a result of the specific PT method or a combination of these factors.

## Introduction

Progression within gymnastics requires the continuous development of new and increasingly complex skills with heightened physical demands, hence for long-term athlete development the importance of strength and power is emphasised [1,2]. Subsequently, training to develop strength and power must begin at a young age in order to achieve maximal potential and complete the required skills for top competitions [3]. It is necessary for gymnasts to possess sufficient explosive power in the lower limb musculature in order to perform the multitude of required jumping skills whilst maintaining body control [4,5]. The handspring vault (HV) is one such manoeuvre which demands considerable force and power output and is of paramount importance for a gymnast’s vaulting development [6]. Although the handspring vault is not performed at high-level competition due to associated low scoring value, it forms the basis of advanced skill acquisition, and is known to serve as a valuable preparation vault for gymnasts and is therefore a pre-requisite for all high quality vaulting [7].

Plyometric training (PT) is a specialised, high-intensity training method which aims to increase sport-specific explosive power and the rate of force development [8]. Plyometric muscle action entails a fast eccentric pre-stretch of the muscle and storage of elastic energy, leading to a powerful explosive concentric contraction, providing greater force and power output than a contraction without the eccentric phase [9]. PT enhances motor unit recruitment and firing frequency and specifically fast twitch fibre recruitment, which leads to enhanced rate and force of muscle contraction [10]. Research shows that PT improves vertical jump performance and injury prevention within adolescent gymnasts [4]. In addition PT has led to improvements in various aspects of power generating ability such as vertical jump performance and short duration sprinting (10 m—40 m) within cohorts of adolescent and junior athletes (13–19 years old) from a range of field and court based team sports [11–15]. In addition PT has been shown to improve swimming block start performance in adolescents [16]. The above findings are pertinent as jumping and sprinting ability are crucial within vaulting. Vaulting features a high velocity approach sprint after which gymnasts aim to perform an effective jump on the springboard to generate power for maximum repulsion off the vault [17].

Despite the considerable demand for strength and power production within gymnastics, there is little investigation of PT directly linked to specific skills in gymnastics [4]. In order to execute skills with maximum control and efficiency it is essential for gymnasts to possess a developed explosive power in both the lower and upper extremity musculature, particularly for vault and tumbling performance [5,18,19]. It is accepted that to reach the elite level in gymnastics requires high levels of strength, power, flexibility and agility and therefore intensive physical training has to begin at a young age [20,21]. Improvements in stretch-shortening cycle function and other performance measures have been reported in youths following short-term PT programs ranging in duration from four to eight weeks [11–14,16]. Subsequently, the aim of this study was to determine the effect of a six-week intervention of PT when added to habitual gymnastic training on handspring vault performance variables and lower body power. It was hypothesised that there would be a significant improvement across numerous HV performance variables and lower body power following completion of a PT intervention.

## Materials and Methods

### Participants

Twenty female gymnasts (Mean ± SD, age: 12.5 ± 1.67 y, stature: 1.46 ± 0.11 m, body mass: 40.5 ± 9.7 kg), with at least three years’ experience in competitive gymnastics were recruited from a local gymnastics club. The participants were all able to execute a HV competently, were regional standard as a minimum, and engaged in at least six hours of gymnastics training a week. Following a health screening questionnaire all participants provided written informed consent and written parental consent was also received for all participants to participate in the study. The study was approved by the School of Sport and Exercise Science ethics committee reviewed and audited by the University of Lincoln Institutional ethics committee in line with the Helsinki Declarations for research with human volunteers.

### Procedures

#### Experimental protocol

Permission to carry out the study and associated procedures was provided by Lincoln Gymnastics Club (Unit 11, Sadler Rd, Lincoln, UK), as this was the location where all experimental procedures took place. Prior to baseline measures, participants engaged in a standardised pre-set 15 min warm up involving running, jumping and stretching. Thereafter, all participants performed three HVs with maximum effort adhering to FIG guidelines [1]. In addition countermovement jump (CMJ) height was assessed using the Just Jump measurement system (Just Jump, Probotics, Huntsville, AL, USA), which served as a measurement of explosive power development [22]. Once the baseline trial was completed, participants were then randomly assigned to either the experimental plyometric training group (PTG), whom performed a PT intervention involving upper and lower body exercises, or the control group (CG). During the six-week intervention period, both groups were advised to continue with their regular habitual gymnastics training. The post-intervention trial took place three days following completion of the intervention to determine the effectiveness of the PT intervention, this involved a reassessment of a further three HV and CMJ height, FIG guidelines were once again adhered to. The CG performed their pre and post testing at the same time points as the PTG.

#### Plyometric training intervention

During the six-week intervention period, both groups followed their habitual gymnastics training ~10 h per week in duration. However, the PTG performed an additional two 40 min PT sessions. The six-week plyometric intervention undertaken by the PTG is depicted in Table 1. Prior to the intervention, the PTG group participants were assessed by an accredited strength and conditioning practitioner on their ability and competency to perform the prescribed exercises to ensure the PT was appropriate and safe. The exercises included in the PT program were selected based upon their specificity to the HV and also adapted from practice and recommendations by Bishop et al [16] and Chimera et al [23]. Particular consideration was placed on ensuring that the exercises and intensity (box–hurdle height) included within the programme provided sufficient muscular overload and adhered to the requirements of safety and welfare of the adolescent participants [16]. Approximately 60 s of rest was provided after each set of exercise. Within each training week the same PT training session was repeated twice. The initial two weeks of the PT featured the performance of 12 to 13 low to moderate intensity plyometric exercises with a moderate overall volume of repetitions. During weeks three and four a wider range of exercises were utilized, including more developed drills and total session volume increased. The final two weeks featured an increased number of repetitions per exercise with more demanding exercises generally selected, the last week featuring a substantial rise in total repetitions volume.

**Table 1:** The six-week sport-specific plyometric intervention used in this study. Denotes sets x repetitions.

Exercise	Week 1	Week 2	Week 3	Week 4	Week 5	Week 6
Tuck jump	2 x 4	2 x 4			4 x 5	4 x 5
Split jump	1 x 4	1 x 4				3 x 6
Squat jump	1 x 4	1 x 4	2 x 4	2 x 4	4 x 5	4 x 5
Jump over barrier (15cm)	2 x 4	2 x 5				
Jump over barrier (30cm)			2 x 4	2 x 4	3 x 4	4 x 4
Alternate leg push off		2 x 4	2 x 4	2 x 5		
Multiple box-to-box jumps			2 x 4	2 x 4	3 x 4	4 x 4
Single leg bounding	1 x 4	1 x 4	2 x 4	3 x 5		
Jump to Box (30cm)			2 x 1	2 x 1	4 x 1	4 x 1
Jump From Box (30cm)			2 x 1	2 x 1	4 x 1	4 x 1
Standing long jump	2 x 1	2 x 1	2 x 1	2 x 1	4 x 1	4 x 1
Bounce to Handstands against wall	1 x 4	1 x 4	2 x 4	2 x 6		
Handstand hops	4 x 1	4 x 1	4 x 1	4 x 1		
Shoulder shrug hops			1 x 4	1 x 4	2 x 4	2 x 4
Chest pass at trampette (Medicine Ball [MB] 2/3kg)	2 x 1	2 x 1	3 x 1	4 x 1		
Single-arm throw at trampette (MB 2/3kg)	2 x 1	2 x 1	3 x 1	4 x 1		
Inverted clap push-ups	2 x 4	2 x 5	3 x 4	1 x 4		
Push-up on and off raised surface				2 x 4	2 x 5	3 x 4
Sit Up Throw (MB 2/3kg)	1 x 4	1 x 4	2 x 4	2 x 5	3 x 4	4 x 4
Total Repetitions	54	66	88	105	106	138

The PT intervention was performed on the vault run (non-compliant concrete floor), enabling rapid use of the stretch-shortening cycle [24]. The PTG were advised to wear sports trainers and sports clothing to reduce injury risk. The PT sessions lasted 60 min in duration comprising of a 10 min warm up before each plyometric session to ensure the musculoskeletal system was activated and prepared [1], a 40 min plyometric intervention and a 10 min cool-down, based upon guidelines from Bishop et al. [16].

#### Handspring vault analysis

The trials took place at a local gymnastics club, using a Gymnova vaulting table. The participants performed a standardised 15 min warm up, involving running jumping and stretching, representing the gymnasts’ competition warm up, and completed two practice vaults, also in line with competition guidelines [1]. The gymnasts then individually prepared for their trial, standing 18 m away from the vaulting table (measured by a tape measure). During the three recordings, a member of the experimental team raised one hand, acknowledging the subject to perform the HV, in accordance to competition standards. The vault was set between 1.10–1.25 m dependent on age, in compliance with competition vault height and remained the same for pre and post HV trials [1].

The HV trials were recorded using two cameras (Casio EX-FH100) at 120 Hz in the sagittal plane of motion. The cameras were set up parallel to the HV motion using a tripod, 6 m away from the vaulting table to the left hand side, and half way down the vault run. Camera one was positioned to allow full viewing of the HV and camera two for the full vault run. Participants were requested to wear leotards, enabling replication of competition and also for accurate analysis.

#### Videography data analysis

**Fig 1:**
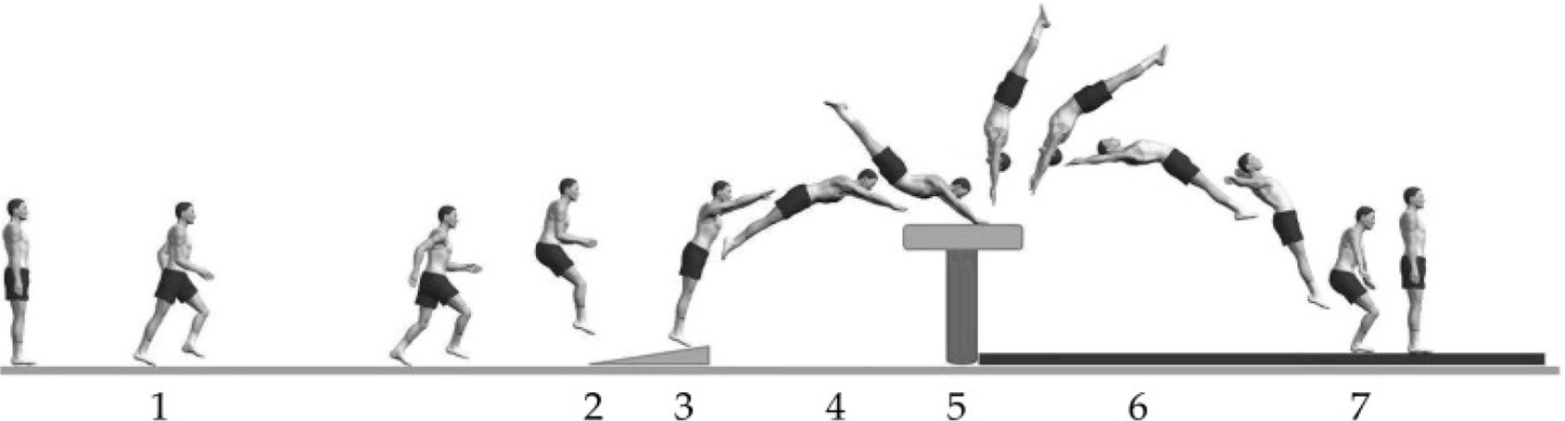
The Seven Phases of the Handspring Vault (1- Approach Run; 2- Take off; 3- Springboard contact; 4- First flight; 5- Repulsion phase; 6- Post Flight; 7- Landing). Adapted from Atiković [25].

Video recordings were uploaded and calibrated into Quintic biomechanical software (Quintic Biomechanics v21 Video Analysis Software, Quintic Consultancy, West Midlands, UK) for analysis of key variables identified for successful HV performance: 1.Run-up velocity (ms-1). 2.Take-off velocity (ms-1) (calculated from the last two steps). 3.Hurdle to board distance (m). 4.Board contact time (s). 5.Pre-flight time (s). 6.Shoulder angle of vaulting table (°). 7.Hip angle on vaulting table (°). 8. Table contact time (s). 9.Post-flight time (s) (Fig 1).

### Statistical Analysis

Statistical analysis was performed using SPSS software (IBM SPSS Statistics, Version 21, IBM UK Limited, Hampshire, UK, 2012). For all variables, data are presented as mean (± SD), unless stated otherwise. A Levene’s test for homogeneity of variance and Sharpio-Wilks test for normality were performed prior to analysis. The significance level was set at *P* < 0.05 for all data analyses. To assess between-group differences, change in mean values for all variables from baseline to intervention trials were calculated and analysed with a two-tailed independent t-test. For data that was not normally distributed, a non-parametric independent sample Mann-Whitney U Test was conducted. To evaluate within-group differences for assessed performance variables a paired-samples t-test was performed for both PTG and CG. However, the take-off velocity data was not normally distributed, therefore a non-parametric Wilcoxon Signed Rank test was performed. In addition effect size (*ES*) was calculated for both within- and between-group data from baseline to post intervention trials corresponding with procedures suggested by Hopkins [26]. In accordance with these procedures interpretation of observed effect sizes are as follows; trivial < 0.2, small 0.2–0.6, moderate 0.6–1.2, large 1.2–2.0, very large > 2.0 [24].

## Results

There were no differences in baseline values on all assessed measures between the PTG and CG (*P* > 0.05). Analysis of the HV variables for the change between pre-post intervention demonstrated significant differences between the PTG and CG. Results indicated a significant difference (*P* = 0.008, *ES* = 0.27 [small]) for post-flight time change score between the PTG compared to CG. Further examination also found a significantly greater change for the PTG than CG for run-up velocity (*P* = 0.002, *ES* = 0.48 [small]), take-off velocity (*P* = 0.005, *ES* = 0.77 [moderate]) hurdle to board distance (*P* = 0.027, *ES* = 0.54 [small]), board contact time (*P* = 0.016, *ES* = 0.49 [small]), table contact time (*P* = 0.029, *ES* = 0.25 [small]) and CMJ height (*P* = 0.037, *ES* = 0.26 [small]). However, no significant difference was found between the PTG and CG for pre-flight time (*P* = 0.67, *ES* = 0.09 [trivial]), shoulder angle on vault (*P* = 0.81, *ES* = 0.04 [trivial]) and hip angle on vault (*P* = 0.14, *ES* = 0.25 [small]). Table 2 displays HV performance variables pre-post intervention for the PTG and CG showing any significant differences between the groups or within-group. Raw data can also be viewed within S1 Table. Raw Results Data.

**Table 2:** Handspring Vault performance variables (mean ± SD) for the experimental plyometric training group and the control group before and after the 6-week plyometric intervention.

	Plyometrics Training Group		Control Group	
Performance Variable	Baseline	Follow up	Baseline	Follow up
Run-up velocity (ms-^1^)	6.54 ± 0.43	6.87 ± 0.42†*	6.72 ± 0.61	6.79 ± 0.58
take-off velocity (ms^-1^)	5.36 ± 0.80	6.07 ± 0.48†*	5.47 ± 0.61	5.65 ± 0.65
Hurdle to board distance (m)	2.18 ± 0.28	2.36 ± 0.26†*	2.24 ± 0.26	2.27 ± 0.28
Pre-flight time (s)	0.26 ± 0.04	0.28 ± 0.04	0.30 ± 0.05	0.30 ± 0.06
Post-flight time (s)	0.43 ± 0.11	0.45 ± 0.10†*	0.43 ± 0.10	0.42 ± 0.10
Board contact time (s)	0.121 ± 0.01	0.115 ± 0.01†*	0.122 ± 0.01	0.122 ± 0.01
Table contact time (s)	0.33 ± 0.08	0.30 ± 0.09†*	0.35 ± 0.09	0.35 ± 0.10
Shoulder angle on vault (°)	154 ± 15	154 ± 13	154 ± 12	153 ± 11
Hip angle on vault (°)	142 ± 19	148 ± 15†	158 ± 18	159 ± 17
Countermovement jump height (cm)	43.5 ± 6.1	45.3 ± 5.8†*	45.1 ± 5.8	45.3 ± 5.5

* = A significant (P < 0.05) difference was observed between pre-intervention and post-intervention trial change scores between-groups.

† = A significant (P < 0.05) difference was observed between pre-intervention and post-intervention trial variables within-groups.

Statistical analysis of within-group differences revealed that the run-up velocity (*P* < 0.001, *ES* = 0.72 [moderate]) and take-off velocity (*P* = 0.001, *ES* = 0.96 [moderate]) was significantly faster after completion of the PT intervention for the PTG, whilst no significant difference was found for the CG (*P* = 0.13, *ES* = 0.13 [trivial]; *P* = 0.051, *ES* = 0.28 [small]). Similarly CMJ height (*P* = 0.021, *ES* = 0.30 [small]) and hurdle to board distance (*P* = 0.009, *ES* = 0.65 [moderate]) were significantly greater for the PTG, whereas no significant differences were found for the CG (*P* = 0.12, *ES* = 0.05 [trivial]; *P* = 0.18, *ES* = 0.14 [trivial]). Differences were also identified in board contact time (*P* = 0.011, *ES* = 0.54 [small]) and table contact time (*P* = 0.014, *ES* = 0.31 [small]) which were significantly lower after the completion of the PT for the PTG. However no differences were present for either board contact time or table contact time for the CG (*P* = 1.00 *ES* = 0.00 [trivial], *P* = 0.23, *ES* = 0.05 [trivial]). Further examination identified, a significant improvement (*P* = 0.022, *ES* = 0.37 [small]) of hip angle on vault for the PTG post intervention, but not for shoulder angle (*P* = 0.65, *ES* = 0.05 [trivial]) on vault. The CG displayed no significant difference for hip angle on vault (*P* = 0.60, *ES* = 0.07 [trivial]) or shoulder angle on vault (*P* = 0.57, *ES* = 0.10 [trivial]) pre-post intervention. Furthermore, there was a significant improvement (*P* = 0.038, *ES* = 0.20 [small]) evident for post-flight time following the PT intervention for the PTG, whilst no significance existed for the CG (*P* = 0.11, *ES* = 0.08 [trivial]). However, no significant difference was found for pre-flight time for either the PTG (*P* = 0.16, *ES* = 0.31 [small]), or the CG (*P* = 0.20, *ES* = 0.16 [trivial]).

## Discussion

The purpose of this study was to investigate the effect of adding a gymnastics-specific PT intervention on HV performance and lower body power development. The principle finding from the investigation showed that the PT intervention increased post-flight time to a ‘small’ but significant degree. This was an important and practically relevant finding since post-flight time has been determined as a key indicator of successful HV performance [1,7] and a marker of athlete preparedness to progress to more challenging vaults [27]. The PTG experienced a significant ‘moderate’ improvement in run-up velocity, which has also been established as another important factor of HV performance. In addition, PTG’s take-off velocity, hurdle to board distance, board contact time, and table contact time underwent ‘small to moderate’ significant improvements following the completion of the six-week PT intervention. However, the CG made no significant improvements in the HV variables pre-post intervention, with any mean changes from pre to post testing being inferred as ‘trivial’ effects [26]. The obtained results confirm the experimental hypothesis that there would be a significant improvement across numerous HV performance variables following completion of the PT intervention. For the HV performance variables that significantly increased following PT the magnitude of effect ranged from ‘small’ to ‘moderate’. However, since the intervention was only six weeks in duration, effects of this magnitude can be seen as practically meaningful [26]. Further portrayed by the consistent between-group differences for post-test performance variables between the PTG and CG.

Significant improvements in run-up velocity and take-off velocity were found after completion of the PT intervention for the PTG, whilst no difference was found for the CG. This supports the findings from previous literature that PT can increase sprint velocity [14], highlighting enhanced force production likely through improved time to initiate muscular contraction and the ability to recruit specific, notably type 2 muscle fibres. However, the findings conflict with Ahmet et al. [11] who found no difference in sprint time following PT. The current study adds to the current literature that vault run-up velocity can be increased through PT, however, it cannot distinguish which stage of the 18 m run-up was mostly affected. In addition, the increase in run-up and take-off velocity and distance from hurdle to board distance all of which were positively enhanced to a ‘moderate’ degree, can likely be attributed to an improvement in the stretch-shortening cycle as a result of PT. The eccentric contraction during plantar flexion of the landing foot and flexion of the knee activates a stretch reflex in the gastrocnemius and quadriceps [23,28]. The concentric contraction immediately follows, allowing the series elastic component to enhance force production [29].

Following completion of the PT intervention the PTG experienced ‘small’ but significant decreases in board contact time and table contact time, demonstrating an improved explosive manoeuvrability within the HV. It can be theorised that this is attributed to improved neuromuscular activation, whereby the ability of the central nervous system to react to a stimulus and generate muscular force has been enhanced [23]. The findings suggest that the PT intervention successfully developed the efficiency of the stretch-shortening cycle, due to a more explosive reaction off the springboard and vault. It is suggested that to efficiently perform powerful movements, the central nervous system elevates motor unit recruitment and firing frequency, subsequently improving the ability to react to stimuli, which is demonstrated in an improved ‘jumping’ and ‘repulsion’ during HV performance after the PT intervention [30]. This finding supports Bishop et al. [16], who found a greater release velocity within the swimming block start, which led to improved performance time following PT.

The improvements found in the HV variables following the PT intervention indicate that a higher HV score may be obtained as a result. However this cannot be fully assumed because not all factors were improved, including shoulder flexion angle and hip extension angle on the vault, mimicking the handstand position on the vault, which is a relevant judging criterion [1]. These technical variables associated to HV may not have been significantly improved since the PT program focuses more on the physiological development of the athlete rather than the technical ability of performing the HV. Nevertheless, the 6-week plyometric program did include activity specific drills (handstand hops and bounce to handstands). This suggests that to improve good handstand position on the vault, focus should be on technique training to stimulate required motor units to enhance technical understanding [27].

CMJ height experienced a ‘small' but significant increase after the PT intervention for the PTG and also in comparison to the CG, with the CG having no significant improvement. This is an important assessment as the CMJ test was used as a standardised measure of power development to assess the effects of PT, since it has previously been found to be enhanced after such interventions involving adolescent and junior participants [11,12,14]. This also contributed to Bishop et al. [16], suggesting that release speed is faster following PT and emphasised that the training group experienced elevated neuromuscular response and force production. Statistical interpretation of the results can assume that PT improves post-flight time, since the PTG significantly increased airborne time in pre-post HV intervention compared to the CG. Moreover, it is important to note that the CG made no improvement in post-flight time. Emphasis was placed on the ability to maintain a fast run-up velocity, and generate a forceful push off the vault, consequently affecting the post-flight time. The ‘small’ improvement in post-flight time highlights how PT can improve the vault, since prolonged flight is indicated in the code of points as a judging criterion [1]. It is the duration of the post-flight where more advanced elements will be added in order to increase vaulting difficulty value, increasing overall score. Consequently, due to the average age of the participants in this study being 12.5 years; the findings show how progression to their gymnastics can be made for the future.

Whilst this study has demonstrated the potential positive effect of adding PT to habitual gymnastics training, it is not possible to be certain that the differences identified are a consequence of the additional volume of sprinting and jumping activities (two hours per week), as a result of the specific PT method or a combination of these factors. Therefore it is recommended that future research examine the effects of similar programming practices that incorporate additional valuating, sprinting and jumping movements vs the effects of PT to determine the underpinning mechanisms of such approaches to training.

When considering and devising plyometric programs for youth and junior athlete’s it is essential that the athletes are assessed on their competency to perform plyometric exercises to ensure correct progression / overload and to avoid injury [2]. The initial development of the program should therefore be low to moderate in intensity and focus upon the technical proficiency of the exercises used to avoid injury [16]. In addition, the exercises should be specific to the movements in which increased power production is sought and should provide a progressive overload through increases in both intensity and volume [4]. Progression to higher overload intensities can be manipulated through altering exercises and increasing the number of repetitions and sets or elevated heights of boxes and hurdles. It is also important to ensure that the PT or power based programs are built around the youth gymnasts training program to provide appropriate recovery periods (24–48 hrs), enabling adaptations to take place and minimise the risk of injury [4, 16].

## Conclusions

This study investigated the effect of a specific PT program on HV performance. The HV is of paramount importance for gymnast’s vaulting development, and includes both technical skill and power production to achieve success. The six-week PT intervention engaged specific muscles used within the HV to employ an effective sport-specific program, improving the force generating capacity and neuromuscular development, which consequently improved aspects of the HV. The results demonstrated that PT caused ‘small to moderate’ significant improvements in run-up velocity, take-off velocity, hurdle to board distance, board contact time, table contact time and post-flight time. As these variables are associated with successful HV performance, it can be suggested that the implementation of a PT program can contribute to improving HV performance and prepare youth gymnasts for the progression of vaulting in the future. However, further research should aim to examine whether the incorporation of additional sprinting, vaulting and jumping movements equal in volume may provide similar benefits to specific PT programs.

## Supporting Information

S1 Table Raw Results Data. (DOC)
