# Supplementary material for: Effect of Plyometric Training on Handspring Vault Performance and Functional Power in Youth Female Gymnasts
Source: PLoS One. 2016 Feb 9;11(2):e0148790. doi: 10.1371/journal.pone.0148790 (PMC4747498; doi:10.1371/journal.pone.0148790)
Supplement: S1 Table — (DOC) [file pone.0148790.s001.doc]

**S1 Table. Raw Results Data**

|  |  | Take-off velocity (ms^-1^) | | Run-up velocity (ms-^1^) | | Hurdle to board distance (m) | | Pre-flight time (s) | | Post-flight time (s) | | Board contact time (s) | |
| --- | --- | --- | --- | --- | --- | --- | --- | --- | --- | --- | --- | --- | --- |
| Participant | Group | Pre | Post | Pre | Post | Pre | Post | Pre | Post | Pre | Post | Pre | Post |
| 1 | EPTG | 6.41 | 6.76 | 7.16 | 7.34 | 2.39 | 2.65 | 0.33 | 0.34 | 0.50 | 0.53 | 0.125 | 0.117 |
| 2 | EPTG | 5.01 | 6.45 | 6.40 | 7.12 | 1.94 | 2.23 | 0.25 | 0.26 | 0.34 | 0.41 | 0.125 | 0.117 |
| 3 | EPTG | 3.43 | 4.89 | 5.97 | 6.22 | 2.75 | 2.71 | 0.29 | 0.29 | 0.53 | 0.53 | 0.108 | 0.108 |
| 4 | EPTG | 5.86 | 6.20 | 7.12 | 7.48 | 2.36 | 2.57 | 0.32 | 0.33 | 0.63 | 0.62 | 0.108 | 0.108 |
| 5 | EPTG | 5.36 | 6.02 | 6.72 | 6.92 | 2.42 | 2.45 | 0.25 | 0.27 | 0.54 | 0.57 | 0.125 | 0.117 |
| 6 | EPTG | 5.62 | 6.13 | 6.03 | 6.34 | 1.97 | 2.41 | 0.25 | 0.26 | 0.30 | 0.31 | 0.100 | 0.100 |
| 7 | EPTG | 5.41 | 5.98 | 6.82 | 6.99 | 1.88 | 1.98 | 0.29 | 0.29 | 0.32 | 0.33 | 0.125 | 0.117 |
| 8 | EPTG | 5.50 | 5.95 | 6.24 | 6.73 | 1.97 | 2.42 | 0.20 | 0.28 | 0.36 | 0.42 | 0.133 | 0.125 |
| 9 | EPTG | 5.98 | 6.12 | 6.72 | 7.07 | 1.97 | 2.01 | 0.25 | 0.23 | 0.40 | 0.39 | 0.142 | 0.125 |
| 10 | EPTG | 5.04 | 6.23 | 6.25 | 6.45 | 2.11 | 2.16 | 0.22 | 0.23 | 0.41 | 0.43 | 0.117 | 0.117 |
| 11 | CG | 5.11 | 5.35 | 6.45 | 6.43 | 2.10 | 2.12 | 0.28 | 0.28 | 0.43 | 0.42 | 0.125 | 0.125 |
| 12 | CG | 5.59 | 6.01 | 6.92 | 7.00 | 2.29 | 2.38 | 0.28 | 0.27 | 0.43 | 0.42 | 0.125 | 0.125 |
| 13 | CG | 5.23 | 5.12 | 6.48 | 6.52 | 1.89 | 1.87 | 0.22 | 0.23 | 0.32 | 0.33 | 0.133 | 0.133 |
| 14 | CG | 5.03 | 5.11 | 6.31 | 6.44 | 1.88 | 1.79 | 0.28 | 0.27 | 0.33 | 0.32 | 0.125 | 0.125 |
| 15 | CG | 5.56 | 5.99 | 6.90 | 7.23 | 2.42 | 2.39 | 0.35 | 0.36 | 0.49 | 0.50 | 0.125 | 0.117 |
| 16 | CG | 5.60 | 5.71 | 5.84 | 6.12 | 2.09 | 2.29 | 0.26 | 0.27 | 0.41 | 0.37 | 0.133 | 0.133 |
| 17 | CG | 5.43 | 6.02 | 7.62 | 7.56 | 2.68 | 2.69 | 0.34 | 0.34 | 0.52 | 0.51 | 0.100 | 0.108 |
| 18 | CG | 6.17 | 6.28 | 7.16 | 7.20 | 2.41 | 2.51 | 0.34 | 0.40 | 0.43 | 0.42 | 0.117 | 0.117 |
| 19 | CG | 6.62 | 6.50 | 7.54 | 7.49 | 2.45 | 2.47 | 0.36 | 0.36 | 0.62 | 0.62 | 0.108 | 0.108 |
| 20 | CG | 4.40 | 4.37 | 5.98 | 5.94 | 2.16 | 2.23 | 0.26 | 0.27 | 0.28 | 0.28 | 0.133 | 0.133 |
| PTG MEAN |  | 5.36 | 6.07 | 6.54 | 6.87 | 2.18 | 2.36 | 0.26 | 0.28 | 0.43 | 0.45 | 0.121 | 0.115 |
| CG MEAN |  | 5.47 | 5.65 | 6.72 | 6.79 | 2.24 | 2.27 | 0.30 | 0.30 | 0.43 | 0.42 | 0.122 | 0.122 |
| PTG SD |  | 0.80 | 0.48 | 0.43 | 0.42 | 0.29 | 0.26 | 0.04 | 0.04 | 0.11 | 0.10 | 0.013 | 0.008 |
| CG SD |  | 0.61 | 0.65 | 0.61 | 0.58 | 0.26 | 0.28 | 0.05 | 0.06 | 0.10 | 0.10 | 0.011 | 0.010 |
|  |  |  |  |  |  |  |  |  |  |  |  |  |  |

|  |  | Table contact time (s) | | Shoulder angle on vault (°) | | Hip angle on vault (°) | | Countermovement jump height (cm) | |
| --- | --- | --- | --- | --- | --- | --- | --- | --- | --- |
| Participant | Group | Pre | Post | Pre | Post | Pre | Post | Pre | Post |
| 1 | EPTG | 0.28 | 0.23 | 159 | 162 | 164 | 164 | 45.9 | 46.8 |
| 2 | EPTG | 0.38 | 0.34 | 155 | 156 | 135 | 149 | 36.5 | 41.7 |
| 3 | EPTG | 0.27 | 0.24 | 150 | 149 | 138 | 149 | 55.3 | 56.0 |
| 4 | EPTG | 0.18 | 0.18 | 170 | 169 | 169 | 169 | 46.5 | 47.9 |
| 5 | EPTG | 0.24 | 0.21 | 172 | 161 | 163 | 163 | 49.5 | 51.3 |
| 6 | EPTG | 0.44 | 0.43 | 170 | 168 | 119 | 123 | 37.2 | 36.9 |
| 7 | EPTG | 0.37 | 0.36 | 144 | 143 | 139 | 142 | 44.7 | 46.3 |
| 8 | EPTG | 0.39 | 0.32 | 158 | 160 | 113 | 134 | 41.3 | 46.4 |
| 9 | EPTG | 0.39 | 0.41 | 129 | 131 | 135 | 137 | 37.0 | 39.2 |
| 10 | EPTG | 0.33 | 0.30 | 137 | 139 | 145 | 150 | 41.6 | 40.6 |
| 11 | CG | 0.32 | 0.30 | 146 | 151 | 158 | 158 | 48.0 | 47.8 |
| 12 | CG | 0.33 | 0.32 | 169 | 165 | 136 | 149 | 46.7 | 46.7 |
| 13 | CG | 0.48 | 0.48 | 134 | 144 | 130 | 127 | 40.9 | 41.9 |
| 14 | CG | 0.43 | 0.43 | 139 | 138 | 163 | 156 | 41.0 | 41.1 |
| 15 | CG | 0.32 | 0.33 | 161 | 161 | 172 | 171 | 38.3 | 39.2 |
| 16 | CG | 0.38 | 0.37 | 152 | 148 | 149 | 150 | 42.2 | 41.7 |
| 17 | CG | 0.29 | 0.29 | 157 | 159 | 169 | 179 | 52.7 | 52.6 |
| 18 | CG | 0.28 | 0.27 | 157 | 150 | 177 | 171 | 40.3 | 41.3 |
| 19 | CG | 0.18 | 0.18 | 173 | 171 | 183 | 179 | 55.9 | 56.0 |
| 20 | CG | 0.49 | 0.48 | 150 | 138 | 139 | 147 | 44.7 | 45.2 |
| PTG MEAN |  | 0.33 | 0.30 | 154 | 154 | 142 | 148 | 43.5 | 45.3 |
| CG MEAN |  | 0.35 | 0.35 | 154 | 153 | 158 | 159 | 45.1 | 45.3 |
| PTG SD |  | 0.08 | 0.09 | 15 | 13 | 19 | 15 | 6.1 | 5.8 |
| CG SD |  | 0.09 | 0.10 | 12 | 11 | 18 | 17 | 5.7 | 5.5 |
